# Pharmacological characterization of the ghrelin receptor mediating its inhibitory action on inflammatory pain in rats

**DOI:** 10.1007/s00726-012-1260-8

**Published:** 2012-03-10

**Authors:** Valeria Sibilia, Francesca Pagani, Emanuela Mrak, Elisa Dieci, Giovanni Tulipano, Francesco Ferrucci

**Affiliations:** 1 Department of Pharmacology, Chemotherapy and Medical Toxicology, Università degli Studi di Milano, Via Vanvitelli, 32, 20129 Milan, Italy; 2 Division of Pharmacology and Toxicology, Department of Biomedical Sciences and Biotechnology, Università degli Studi di Brescia, Brescia, Italy; 3 Department of Veterinary Clinical Sciences, Università degli Studi di Milano, Milan, Italy

**Keywords:** Ghrelin receptors, Desacyl-ghrelin, Hyperalgesia, Edema, Randall and Selitto

## Abstract

Recent research suggests a role for ghrelin in the modulation of inflammatory disorders. However, the type of ghrelin receptor (GHS-R) involved in both the anti-inflammatory and anti-hyperalgesic actions of ghrelin remains to be characterized. In this study, we examined whether the inhibitory effect of ghrelin in the development of hyperalgesia and edema induced by intraplantar carrageenan administration depends on an interaction with GHS-R1a. Both central (1 nmol/rat, i.c.v.) and peripheral (40 nmol/kg, i.p.) administration of the selective GHS-R1a agonist EP1572 had no effect on carrageenan-induced hyperalgesia measured by Randall–Selitto test and paw edema. Furthermore, pre-treatment with the selective GHS-R1a antagonist, d-lys^3^-GHRP-6 (3 nmol/rat, i.c.v.) failed to prevent the anti-hyperalgesic and anti-inflammatory effects exerted by central ghrelin administration (1 nmol/rat), thus indicating that the type 1a GHS-R is not involved in these peptide activities. Accordingly, both central (1 and 2 nmol/rat, i.c.v.) and peripheral (40 and 80 nmol/kg, i.p.) administration of desacyl-ghrelin (DAG), which did not bind GHS-R1a, induced a significant reduction of the hyperalgesic and edematous activities of carrageenan. In conclusion, we have shown for the first time that DAG shares with ghrelin an inhibitory role in the development of hyperalgesia, as well as the paw edema induced by carrageenan and that a ghrelin receptor different from type 1a is involved in the anti-inflammatory activities of the peptide.

## Introduction

In this second decade of ghrelin research, numerous studies showed considerable complexity within the ghrelin/ghrelin receptor axis and indicate that an exciting aspect of ghrelin biology could be the identification of receptors subtypes mediating the various functions of the ghrelin-family peptides.

Ghrelin, the endogenous ligand for the growth hormone secretagogue receptor (GHS-R), was discovered in 1999 (Kojima et al. 1999). Ghrelin derives from a 117 amino acid pre-prohormone which is cleaved into a 94 amino acid proghrelin peptide. This proghrelin peptide is further cleaved and gives rise to the 28 amino acid ghrelin peptide. Two major forms of 28 amino acid ghrelin have been identified (Hosoda et al. 2000). The ghrelin peptide acylated at serine-3 by ghrelin-*O*-acyltransferase (GOAT) (Gutierrez et al. 2008; Yang et al. 2008), usually referred as ghrelin in the literature, and a non-acylated form (DAG) which circulates at tenfold higher levels than ghrelin (Holmes et al. 2009; Patterson et al. 2005).

In addition to its GH-releasing activity, ghrelin influences a broad range of biological processes, such as food intake and energy expenditure, cell proliferation, gastrointestinal, cardiovascular, pancreatic, pulmonary and immune functions (Cummings 2006; Leite-Moreira and Soares 2007; Van der Lely et al. 2004). The biological activities exerted by ghrelin seem to be mediated by an interaction with specific receptors. Two forms of GHS-Rs have been identified: the fully functional type 1a (GHS-R1a) and the truncated splice variant GHS-R1b (Muccioli et al. 2001). The physiological role of GHS-R1b is not clear, even if in vitro studies have suggested that GHS-R1b may act as a negative regulator of GHS-R1a, thus reducing its constitutive activation (Leung et al. 2007). The octanoylation is critical for the binding to the GHS-R1a and in inducing GH secretion, food intake (Kojima and Kangawa 2005) and inhibition of gastric acid secretion (Sibilia et al. 2006b). In fact, DAG seems to be devoid of any endocrine and gastrointestinal activities.

Interestingly, both ghrelin and DAG inhibit apoptosis of cardiomyocytes and endothelial cells (Baldanzi et al. 2002), which do not express GHS-R1a suggesting the existence of an alternative, functionally active binding site, yet to be identified (Seim et al. 2011), which could mediate some of the effects of both peptides.

Recently, a number of studies have focussed on the role of ghrelin in the control of pain perception. This interest is justified by the evidence that the expression of ghrelin was found in various brain areas involved in the control of nociception, such as the hypothalamus, the sensorimotor area of the cortex, the midbrain and the spinal cord (Guan et al. 1997; Hou et al. 2006; Vergnano et al. 2008; Zigman et al. 2006). The expression of GHS-R mRNA in the brain is in keeping with the widespread distribution of ghrelin receptors. Ghrelin receptors, in fact, are expressed in the hypothalamus, in the pons medulla oblongata, in the substantia nigra and dorsal and median raphe nuclei, regions implicated in the control of pain transmission (Nakazato et al. 2001; Zigman et al. 2006).

Supporting a role for ghrelin as a pain modulator is also the reported interaction of the peptide with endogenous opioid containing neurons in the hypothalamic arcuate nucleus and endocannabinoid system known to exert a modulatory role in the central and peripheral regulation of pain perception (Bloom et al. 1978; Riediger et al. 2003; Tucci et al. 2004).

It has been reported that ghrelin administered either centrally or peripherally prevents the development of acute hyperalgesia induced by intraplantar carrageenan injection in the rat (Sibilia et al. 2006a), and that long-term treatment with ghrelin attenuates chronic neuropathic pain (Guneli et al. 2010). Furthermore, ghrelin has been shown to modulate inhibitory transmission in deep mouse spinal cord dorsal horn (Ferrini et al. 2009; Vergnano et al. 2008).

In addition to an anti-nociceptive activity, ghrelin seems to be a potent anti-inflammatory mediator. Strengthening this view are in vitro studies showing that ghrelin is able to inhibit the expression of the pro-inflammatory cytokines IL-1β, IL-6 and TNFα by T-cell receptor-activated T cells, LPS-activated monocytes and dendritic cells (Dixit et al. 2004, Taub 2007) and to reduce pro-inflammatory responses and nuclear factor κB activation in endothelial cells (Li et al. 2004).

In vivo studies have shown that ghrelin treatment reduces the severity of experimental models of sepsis (Dixit et al. 2004; Wu et al. 2005), colitis (Gonzalez-Rey et al. 2006) and paw edema consequent to carrageenan injection (Sibilia et al. 2006a). Plus, long-term treatment with the synthetic GHS-R agonist, GHRP-2 attenuates arthritis in a rat model (Granado et al. 2005).

Also, clinical trials support the possibility that ghrelin administration may be beneficial for cachexic patients and for patients with chronic inflammatory diseases (Ashitani et al. 2009; Laviano et al. 2010). Such findings outline the potential therapeutic use of ghrelin in inflammatory disorders.

However, the type of ghrelin receptor involved in both the anti-hyperalgesic and anti-inflammatory activities of the peptide remains to be clarified. The aim of this study was, therefore, to examine whether both the anti-hyperalgesic and anti-inflammatory activities of ghrelin depend on an interaction with GHS-R1a.

For this purpose, we examined the effects of intracerebroventricular (i.c.v.) or peripheral (i.p.) injection of the GHS-R1a agonist, EP1572 (Broglio et al. 2002; Sibilia et al. 2006b) on carrageenan-induced hyperalgesia and paw edema and the reversibility of both the ghrelin anti-nociceptive and anti-inflammatory activities by the specific GHS-R1a antagonist d-lys^3^-GHRP-6 (Carreira et al. 2006; Muccioli et al. 2007; Sibilia et al. 2006b).

Carrageenan is a sulfated polysaccharide extracted from the seaweed *Chondrus crispus*. Carrageenan-induced hind paw inflammation is a neutrophil-mediated acute inflammatory response with clinical symptoms peaking at 1½ to 3 h after the intraplantar injection of carrageenan (Winter et al. 1962). The carrageenan-inflamed hind paw also is painful, and hyperalgesia can be reliably measured using established behavioral pain assays such as Randall and Selitto test (Randall and Selitto 1957).

On the basis of the results obtained, we examined the effect of central or peripheral injection of DAG, which does not bind the classic GHS-R1a (Bednarek et al. 2000; Ghigo et al. 2005), on pain perception and paw edema by using the same experimental model of acute inflammation.

## Methods

### Animals and surgery

Male Sprague–Dawley rats (125–150 g; Harlan Italy) were used. Upon arrival, the rats were housed in single cages under controlled illumination (12 h light/12 h darkness cycle), humidity (65%) and temperature (22 ± 2°C) with free access to water and laboratory chow pellets (Charles River).

For the i.c.v. administration of peptides, a polyethylene cannula (PE10) was implanted into the left lateral ventricle of the brain, 5 days before the experiment, as previously described (Netti et al. 1984). At the end of the experiment (after rats were euthanized with CO_2_), 5 μl of dye (0.5% Evans blue) was injected through the cannula to confirm its position in the ventricle.

All experiments were approved by the Italian Ministry of Health and performed in accordance with the European Directive 2010/63/EU.

### Drugs

Ghrelin, DAG and EP1572 [Aib-DTrp-DgTrp-CHO] were synthesized by conventional solid phase synthesis and purified to at least 98% by HPLC by Neosystem (Strasburg, France). d-Lys^3^-GHRP-6 was purchased from Bachem AG (Budendorf, Switzerland). The peptides were dissolved in saline immediately before the experiment and were injected in a volume of 5 μl/rat, i.c.v. or 2 ml/kg, i.p. carrageenan (0.1 ml of 1% solution in distilled water, Gianni, Italy), which was injected into the right plantar hind paw (i.pl.). In all experiments, an equal volume of saline was used as control.

### Carrageenan-induced hyperalgesia and edema

The nociceptive thresholds of the hind paw after mechanical stimulation, according to the method of Randall and Selitto (1957), were quantified with a Basile algesimeter (Milan, Italy). Each rat was gently held with a hind paw placed under a pressure pad. The force, in grams, applied to the paw was increased at a constant rate until the rat withdrew its paw. The pressure was immediately removed and the force required to elicit the end-point response was noted. A cutoff of 400 g was used. The rats were trained in the nociceptive test daily for 2 days before the experiment. This adaptation procedure produces a stable baseline paw pressure threshold (PPT) (Taiwo et al. 1989).

Carrageenan-induced paw edema was measured by water displacement plethysmography at selected times after carrageenan injection by placing the paw up to the tibio-tarsic articulation (marked with ink) into the chamber of the plethysmometer (Basile, Milan, Italy). The displacement volume (ml) was measured by a transducer.

### Experimental procedures

On the day of the experiment, following baseline testing, carrageenan (*T*_0_) was injected into the plantar region (i.pl.) of the rat right hind paw. The GHS-R1a agonist, EP1572, DAG and ghrelin were injected by the i.c.v route 5 min before carrageenan. When peripherally administered, EP1572 and DAG were injected 30 min before carrageenan. We used doses of EP1572 or DAG equimolar to those of ghrelin previously reported to exert an anti-inflammatory activity (Sibilia et al. 2006a).

To see whether the GHS-R1a antagonist, d-lys^3^-GHRP-6, was able to interfere with the central anti-hyperalgesic and anti-inflammatory effect of ghrelin, the compound was injected i.c.v. 5 min before ghrelin (1 nmol/rat, i.c.v.) and 135 min from carrageenan. d-Lys^3^-GHRP-6 was administered at a dose previously reported to abolish the inhibitory effect of ghrelin on gastric acid secretion (Sibilia et al. 2006b).

In all cases, the nociceptive thresholds and the volume of the inflamed paw were measured before (basal) and at 150, 210 and 270 min after carrageenan. The operator of the paw tests was unaware of the treatments during the test period.

### Statistical analysis

Statistical analysis was performed by a statistical package (SigmaStat, vers. 3.1 Systat Software). All data are reported as the mean ± SEM. Data were analyzed by two-way repeated measures analysis of variance (ANOVA, factors: treatment and time) followed by Bonferroni *t* test. A probability of *P* < 0.05 was considered to be significant.

## Results

### Effects of central or peripheral administration of the GHS-R1a agonist, EP1572, on carrageenan-induced hyperalgesia and edema

**Fig. 1:**
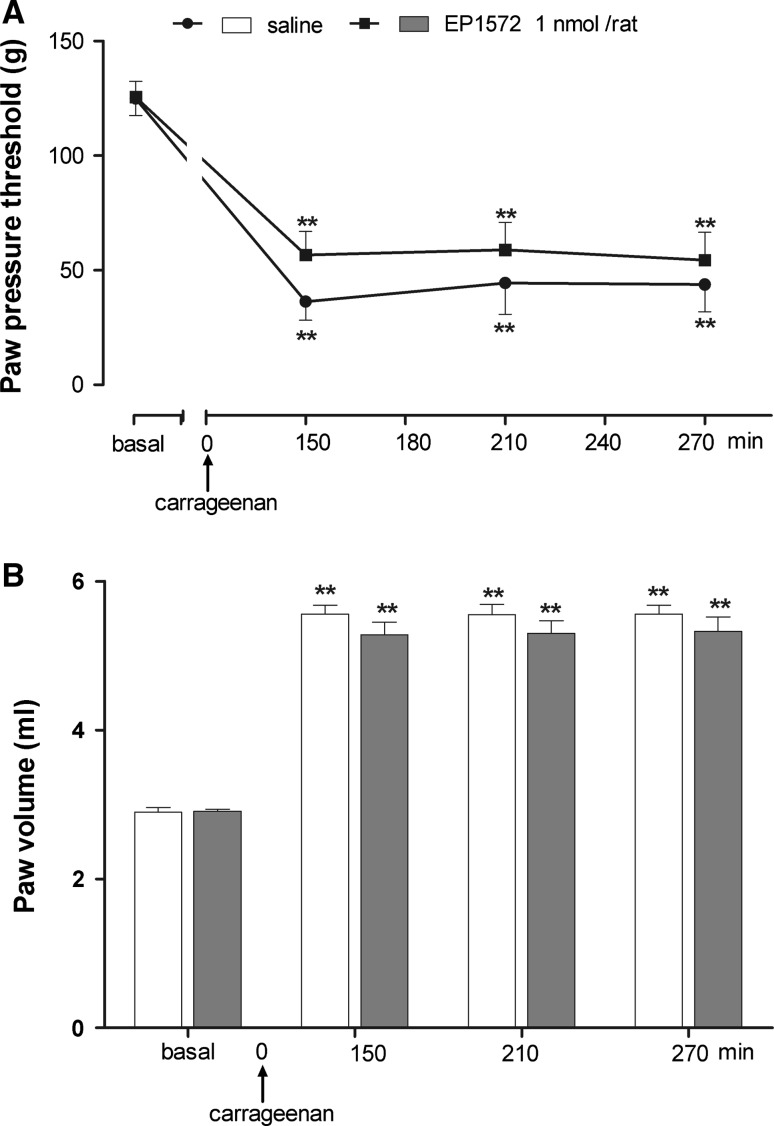
Effect of intracerebroventricular (i.c.v.) injection of EP1572 on hyperalgesia (**a**) and paw edema (**b**) induced by carrageenan. EP1572 was administered 5 min before i.pl. carrageenan. Paw pressure threshold and paw volume were measured prior to carrageenan (basal) and at various times after carrageenan. Each value is the mean ± SEM of 10–12 rats. **P < 0.001 versus basal

As shown in Fig. 1a and b, i.pl. injection of carrageenan produced a significant reduction of PPT and an increase in paw volume as compared with the pre-carrageenan values. Central administration of 1 nmol/rat of the GHS-R1a agonist, EP1572, had no significant effect on carrageenan-induced hyperalgesia (*F*_treatment_ = 0.241; *F*_time_ = 38.25; *P* < 0.001) and paw edema (*F*_treatment_ = 0.16; *F*_time_ = 271.65; *P* < 0.001).

**Fig. 2:**
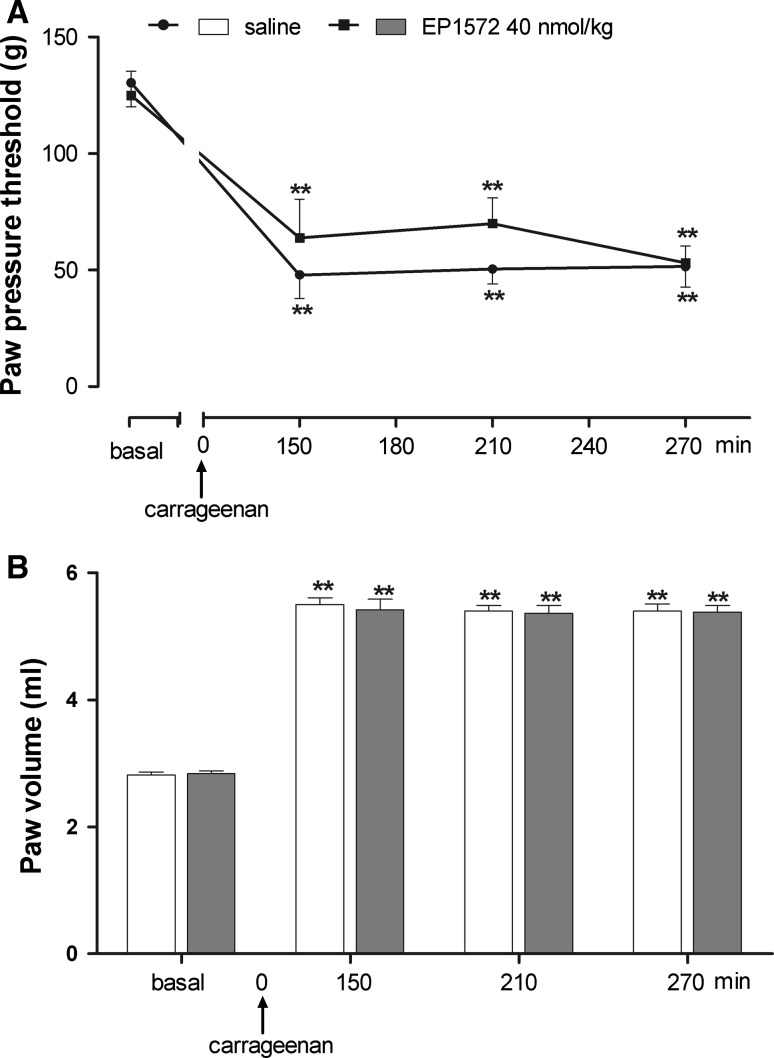
Effect of peripheral (i.p.) injection of EP1572 on hyperalgesia (**a**) and paw edema (**b**) induced by carrageenan. EP1572 was administered 30 min before i.pl. carrageenan. Paw pressure threshold and paw volume were measured prior to carrageenan (basal) and at various times after carrageenan. Each value is the mean ± SEM of 10–12 rats. **P < 0.001 versus basal

Similar results on carrageenan-induced hyperalgesia (*F*_treatment_ = 0.85; *F*_time_ = 42.36; *P* < 0.001) and paw edema (*F*_treatment_ = 0.094; *F*_time_ = 607.43; *P* < 0.001) were obtained when EP1572 (40 nmol/kg, i.p.) was peripherally administered 30 min prior to carrageenan (Fig. 2a, b).

### Effects of central administration of the GHS-R1a antagonist, d-lys^3^-GHRP-6, on ghrelin-induced anti-nociceptive and anti-inflammatory activities

**Fig. 3:**
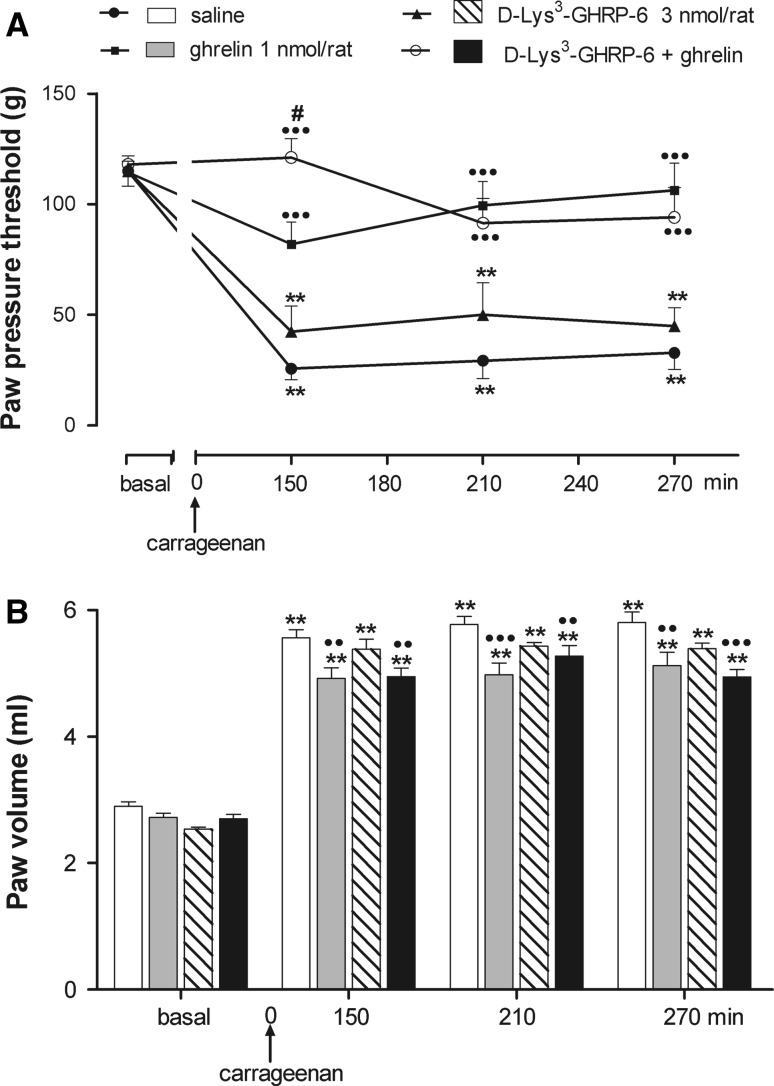
Effect of pretreatment (5 min before) with d-lys^3^-GHRP-6 on the inhibitory action of ghrelin on hyperalgesia (**a**) and paw edema (**b**) induced by i.pl. carrageenan. Ghrelin was injected i.c.v. 5 min before i.pl. carrageenan. Paw pressure threshold and paw volume were measured prior to carrageenan (basal) and at various times after carrageenan. Each value is the mean ± SEM of 12–14 rats. **P < 0.001 versus basal; ^••^ *P* < 0.01, ^•••^ *P* < 0.001 versus saline; ^**#**^ *P* < 0.05 versus ghrelin

As expected, central ghrelin administration (1 nmol/rat, i.c.v.) caused a significant inhibition of carrageenan-induced hyperalgesia as compared with saline-treated rats, which peaked at 150 min from carrageenan and lasted until 270 min. Central administration of the specific GHS-R1a antagonist, d-lys^3^-GHRP-6 (3 nmol/rat, i.c.v.), had no effect on the development of hyperalgesia induced by carrageenan. When administered before ghrelin, d-lys^3^-GHRP-6 not only did not affect the anti-hyperalgesic effect of central ghrelin but significantly increased the anti-hyperalgesic effect of ghrelin at 150 min from carrageenan (Fig. 3a). Two-way ANOVA revealed significant main effects of treatment (*F* = 29.85; *P* < 0.001), time (*F* = 21.57; *P* < 0.001) and interaction between treatment and time (*F* = 4.22; *P* < 0.001).

d-Lys^3^-GHRP-6 had no effect on carrageenan-induced paw edema and failed to remove the anti-inflammatory action of ghrelin (Fig. 3b). In fact, the reduction of paw edema in rats treated with d-lys^3^-GHRP-6 and ghrelin was similar to that of ghrelin treated rats. Two-way ANOVA revealed significant main effects of treatment (*F* = 7.29; *P* = 0.001), time (*F* = 555.16; *P* < 0.001) and interaction between treatment and time (*F* = 2.53; *P* = 0.01).

**Fig. 4:**
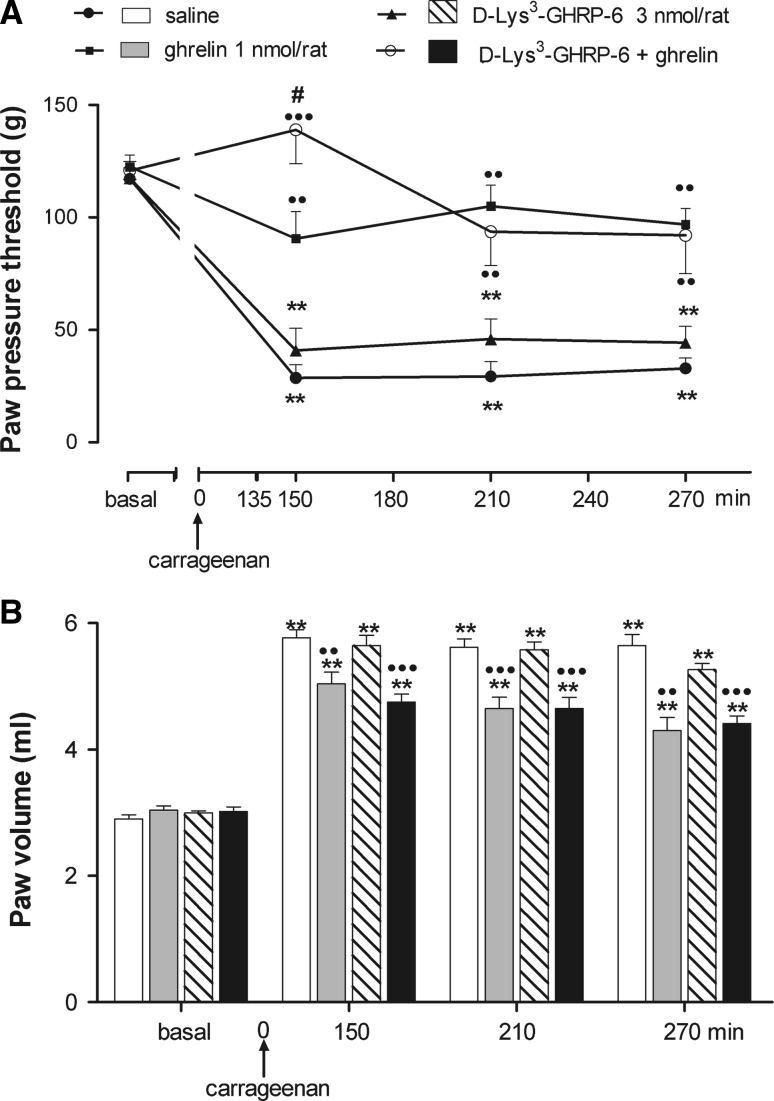
Effect of intracerebroventricular (i.c.v.) injection of d-lys^3^-GHRP-6 on the inhibitory action of ghrelin on hyperalgesia (**a**) and paw edema (**b**) induced by i.pl. carrageenan. d-Lys^3^-GHRP-6 was injected 5 min before ghrelin and 135 min after carrageenan. Ghrelin was injected i.c.v. 5 min before i.pl. carrageenan. Paw pressure threshold and paw volume were measured prior to carrageenan (basal) and at various times after carrageenan. Each value is the mean ± SEM of 6–8 rats. **P < 0.001 versus basal; ^••^ *P* < 0.01, ^•••^ *P* < 0.001 versus saline; ^**#**^ *P* < 0.05 versus ghrelin

To test the possibility that the ineffectiveness of d-lys^3^-GHRP-6 could be due to its short duration of action, rats were treated i.c.v. with d-lys^3^-GHRP-6 (3 nmol/rat, i.c.v.) twice, before ghrelin injection (1 nmol/rat, i.c.v.) and 135 min after carrageenan administration (Fig. 4a, b). Also in this case, d-lys^3^-GHRP-6 was not able to remove the anti-hyperalgesic (*F*_treatment_ = 7.4; *P* = 0.001; *F*_time_ = 14.86; *P* < 0.001; *F*_treatment and time_ = 2.51; *P* = 0.01) and anti-inflammatory (*F*_treatment_ = 15.34; *P* < 0.001; *F*_time_ = 329.17; *P* < 0.001; *F*_treatment and time_ = 6.64; *P* < 0.001) effects of central ghrelin administration.

### Effects of central or peripheral administration of DAG on carrageenan-induced hyperalgesia and edema

**Fig. 5:**
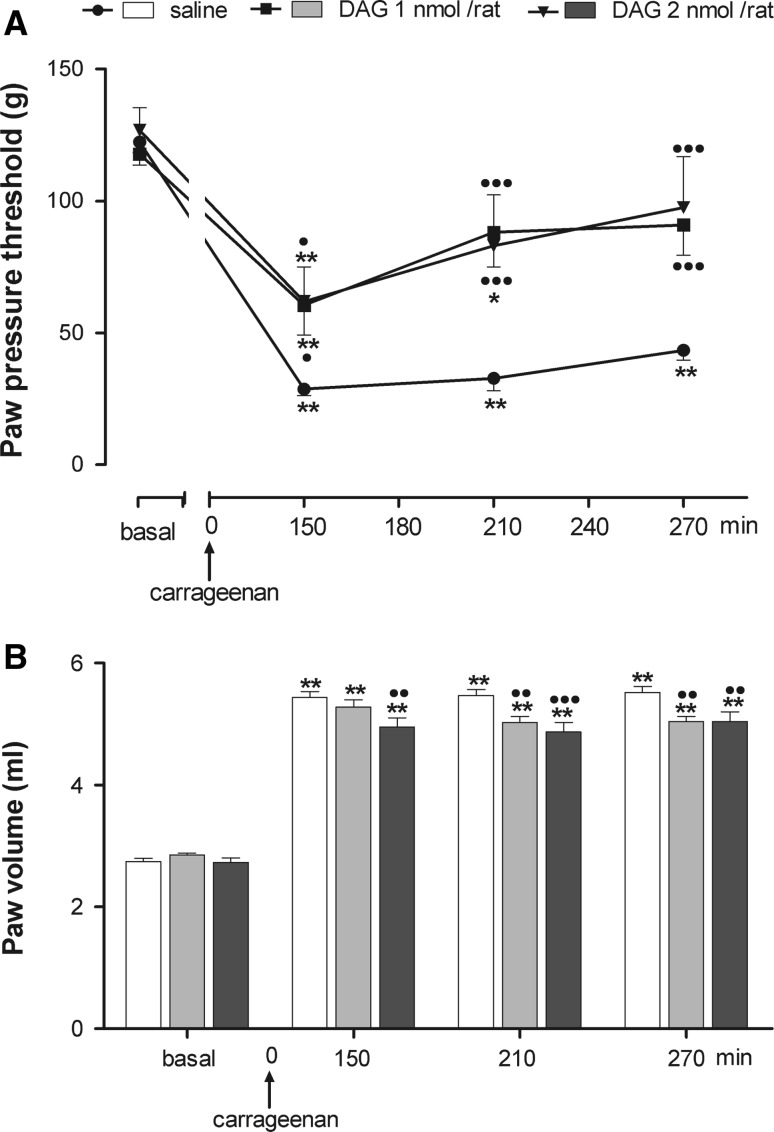
Effect of intracerebroventricular (i.c.v.) injection of desacyl-ghrelin (DAG) on hyperalgesia (**a**) and paw edema (**b**) induced by carrageenan. DAG was administered 5 min before i.pl. carrageenan. Paw pressure threshold and paw volume were measured prior to carrageenan (basal) and at various times after carrageenan. Each value is the mean ± SEM of 10–12 rats. *P < 0.05, **P < 0.001 versus basal; ^•^ *P* < 0.05, ^••^ *P* < 0.01, ^•••^ *P* < 0.001 versus saline

To test the possibility that the anti-nociceptive activity of ghrelin could be linked to an interaction with a receptor different from GHS-R1a, we treated rats either i.c.v. or i.p. with DAG, which is devoid of effects linked to GHS-R1a activation (Kojima and Kangawa 2005; Seim et al. 2011). Figure 5a shows that central DAG administration caused a significant reduction in the hyperalgesia induced by carrageenan starting from 150 min after carrageenan throughout the experimental period. The anti-nociceptive effects observed at the doses of 1 and 2 nmol/rat were of the same intensity. Two-way ANOVA revealed significant main effects of treatment (*F* = 10.6; *P* < 0.001), time (*F* = 49.12; *P* < 0.001) and interaction between treatment and time (*F* = 4.13; *P* < 0.01).

DAG anti-nociception was accompanied by an anti-inflammatory activity. DAG at 1 and 2 nmol/rat induced a long-lasting decrease of paw edema that reaches statistical significance from carrageenan–saline-treated rats starting from 210 and 150 min from carrageenan, respectively (Fig. 5b). Two-way ANOVA revealed significant main effects of treatment (*F* = 5.43; *P* < 0.01), time (*F* = 721.22; *P* < 0.001) and interaction between treatment and time (*F* = 5.55; *P* < 0.01).

**Fig. 6:**
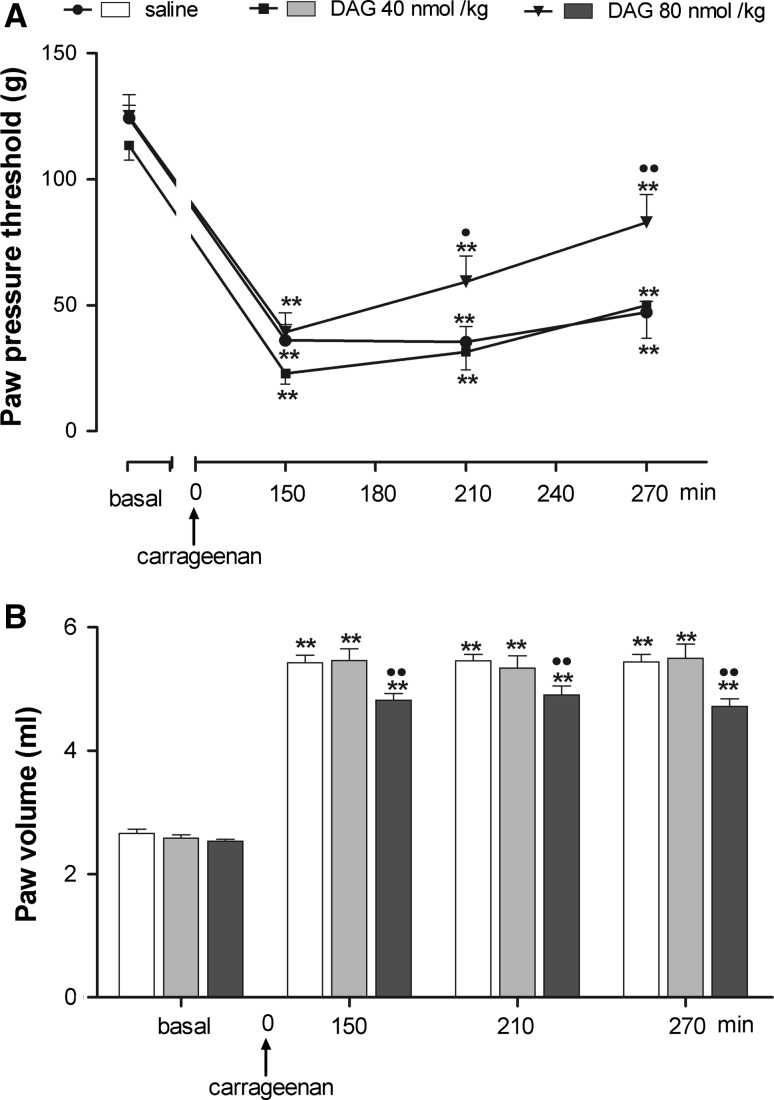
Effect of peripheral (i.p.) injection of desacyl-ghrelin (DAG) on hyperalgesia (**a**) and paw edema (**b**) induced by carrageenan. DAG was administered 30 min before i.pl. carrageenan. Paw pressure threshold and paw volume were measured prior to carrageenan (basal) and at various times after carrageenan. Each value is the mean ± SEM of 10–12 rats. **P < 0.001 versus basal; ^•^ *P* < 0.05, ^••^ *P* < 0.01 versus saline

Systemic DAG administration (40 and 80 nmol/kg, i.p) induced a significant increase in PPT peaking at 270 min from carrageenan, only at the higher dose used (Fig. 6a). Two-way ANOVA revealed significant main effects of treatment (*F* = 8.1; *P* < 0.002) and time (*F* = 89.58; *P* < 0.001).

DAG at 80 nmol/kg i.p. induced a significant decrease of paw edema compared with carrageenan–saline-treated rats starting from 150 min from carrageenan throughout the experimental period (Fig. 6b). Two-way ANOVA revealed significant main effects of treatment (*F* = 5.23; *P* < 0.01), time (*F* = 533.41; *P* < 0.001) and interaction between treatment and time (*F* = 2.25; *P* = 0.05).

## Discussion

The present study was aimed to determine whether GHS-R1a mediates the inhibitory effects of ghrelin on hyperalgesia and paw edema induced by carrageenan in the rat.

For this purpose, we used ghrelin, synthetic analogues acting as GHS-R1a agonist (EP1572) or antagonist (d-lys^3^-GHRP-6) and the natural ghrelin isoform, DAG which does not bind GHS-R-1a (Muccioli et al. 2007).

The results obtained demonstrate that either central or peripheral EP1572 administration did not reduce the development of hyperalgesia induced by i.pl. carrageenan. Furthermore, pre-treatment with the selective GHS-R1a antagonist, d-lys^3^-GHRP-6 did not prevent the inhibitory control of inflammatory pain elicited by central ghrelin administration.

These observations raise the possibility that the anti-nociceptive action of ghrelin could be independent of the presently known ghrelin receptor GHS-R1a. Accordingly, both central and peripheral DAG administration was effective in preventing carrageenan-induced hyperalgesia.

It is possible that the anti-hyperalgesic effect of DAG could be due to a modulatory action on the central pain circuits activated by peripheral inflammation. Supporting this view are the present data showing that a high dose of peripherally administered DAG (80 nmol/kg) is required to achieve an anti-hyperalgesic effect similar to the one induced by central DAG injection (1 nmol/rat) and by previous studies demonstrating that DAG is able to cross the BBB and to increase c-Fos expression in the arcuate nucleus and in the paraventricular nucleus of the hypothalamus (Chen et al. 2005).

When we compared the anti-hyperalgesic effects of ghrelin and DAG, we found that the intensity and pattern of DAG response differ from those of ghrelin.

Ghrelin, in fact, centrally or peripherally administered exerts a dose-dependent and long-lasting anti-nociceptive activity that peaked at 150 min from carrageenan (Sibilia et al. 2006a), whereas DAG, administered at doses equimolar to the ones previously used for ghrelin, elicits a less marked anti-nociceptive activity that peaked at 270 min from carrageenan.

The differences in the onset and the anti-hyperalgesic activity between ghrelin and DAG could imply that DAG must be acylated and converted into the active octanoylated form by the recently identified enzyme GOAT (Gutierrez et al. 2008; Yang et al. 2008). To understand the physiological role of acylated ghrelin and the relevance of the conversion of DAG into the acylated form in the control of pain perception, further studies with GOAT knockout mice or with the pharmacological GOAT antagonist, Go-CoA-Tat (Barnett et al. 2010), will be required.

Alternatively, DAG could recognize, with lower affinity than ghrelin, a common binding site different from GHS-R1a involved in the modulation of pain perception. In this context, binding studies have shown that DAG is able to displace [^125^I]-Tyr^4^-ghrelin binding from cells which do not express GHS-R1a (Muccioli et al. 2007).

The lack of the involvement of GHS-R1a in mediating the anti-nociceptive action of ghrelin is supported by the present results obtained with the selective GHS-R1a agonist (EP1572), which has no effect on carrageenan-induced hyperalgesia. It is unlikely that the lack of effect of peripherally administered EP1572 could be due to its pharmacokinetic profile, since we used a dose of EP1572 higher than those previously reported to be effective in inhibiting gastric acid secretion in rats 3 h after pylorus ligation (Sibilia et al. 2006b).

The anti-hyperalgesic action of ghrelin was not blocked by pre-treatment with the specific GHS-R1a antagonist, d-lys^3^-GHRP-6. It is worth noting that in rats pre-treated with d-lys^3^-GHRP-6, ghrelin induced at 150 min from carrageenan, an increase in PPT was significantly higher than the one detected in rats receiving ghrelin alone. Thus, it is possible that d-lys^3^-GHRP-6 by acting as an antagonist for GHS-R1a could have shifted ghrelin binding to the receptor type mainly involved in the control of pain perception.

At variance to our results, Vergnano et al. (2008) have shown that ghrelin enhances inhibitory neurotransmission in the mouse spinal cord dorsal horn mainly through an interaction with GHS-R1a. However, the authors did not rule out the existence of an unknown additional ghrelin receptor different from the GHS-R1a mediating the anti-nociceptive action of the peptide. In fact, d-lys^3^-GHRP-6 was not able to completely remove the ghrelin inhibitory effect, and a subset of ghrelin responsive neurons showed an increase in the frequency of spontaneous inhibitory postsynaptic currents after DAG application.

It may well be possible that the varying results of these studies could depend on the different experimental models (in vitro vs. in vivo studies) or to the different animal species used (mouse vs. rat). However, further in vivo studies designed to examine the effects of intrathecal ghrelin will be necessary to clarify the involvement of spinal GHS-R1a in modulating spinal nociceptive transmission.

It is unlikely that the GHS-R1a could be involved in the central anti-inflammatory action of ghrelin, since we found that central EP1572 injection has no effect on carrageenan-induced edema and that pre-treatment with d-lys^3^-GHRP-6 failed to modify the central anti-edematous action of ghrelin.

Support for an anti-inflammatory role for peripheral GHS-R1a comes from several studies. In vitro studies have shown that GHS-R1a is expressed on lymphocytes and that administration of a GHS-R1a agonist decreases expression of inflammatory cytokines by activated monocytes and T cells (Dixit et al. 2004). Furthermore, long-term administration of the synthetic GHS-R1a agonist, GHRP-2 has an anti-inflammatory effect in arthritic rats (Granado et al. 2005).

The involvement of peripheral GHS-R1a in the inhibitory role of ghrelin on the development of acute inflammation induced by carrageenan seems to be ruled out. In fact, neither intraplantar ghrelin injection (Sibilia et al. 2006a) nor peripheral administration of the GHS-R1a agonist EP1572 was able to modify paw edema induced by carrageenan. These discrepancies could be linked to differences related to the rodent models of inflammation used or to the dose and schedule of ghrelin/synthetic GHS treatment.

Interestingly, we found that DAG shares with ghrelin a central anti-inflammatory activity. However, also in this case, DAG was less effective than ghrelin reaching the maximal decrease in paw edema at a dose (2 nmol/rat) higher than that previously shown for ghrelin (1 nmol/rat).

Recent in vitro studies have suggested that the scavenger receptor CD36 could be involved in the anti-inflammatory action of DAG. In fact, DAG is able to reduce the β-amyloid activation of CD36 in mouse microglia cells which results in increased release of reactive oxygen species and inflammatory cytokines, whereas ghrelin was inactive (Bulgarelli et al. 2009).

The possibility that CD36 could be involved in the in vivo anti-inflammatory activity of DAG seems to be ruled out, since hexarelin, a synthetic GHS which specifically binds to CD36 (Muccioli et al. 2007) and shares with DAG the ability to interfere with activation of CD36 in microglia cells (Bulgarelli et al. 2009), has no significant effect both on carrageenan-induced hyperalgesia and paw edema (data not shown).

In conclusion, we have shown for the first time that DAG shares with ghrelin an inhibitory role in the development of hyperalgesia, as well as the paw edema induced by carrageenan and that a ghrelin receptor different from 1a is involved in both the anti-inflammatory and anti-hyperalgesic activities of the peptide. This assumption is supported by previous studies indicating that DAG, did not bind to GHS-R1a and by the present results showing that the selective GHS-R1a agonist, EP1572, was ineffective in reducing the development of inflammation induced by carrageenan. Furthermore, the GHS-R1a antagonist, d-lys^3^-GHRP-6 did not alter both the anti-hyperalgesic and anti-inflammatory action of ghrelin.

The identification of natural or synthetic ligands acting with high specificity on this type of ghrelin receptor would have perspective in terms of clinical application as anti-inflammatory drugs.
